# Risk factors, quality of care and prognosis in South Asian, East Asian and White patients with stroke

**DOI:** 10.1186/1471-2377-13-74

**Published:** 2013-07-05

**Authors:** Nadia A Khan, Hude Quan, Michael D Hill, Louise Pilote, Finlay A McAlister, Anita Palepu, Baiju R Shah, Limei Zhou, Hong Zhen, Moira K Kapral

**Affiliations:** 1 Department of Medicine, University of British Columbia, 620 B, 1081 Burrard Street, St. Paul’s Hospita, Vancouver, BC V6Z 1Y6, Canada; 2 Department of Community Health Sciences, University of Calgary, Calgary, AB, Canada; 3 Department of Clinical Neurosciences, University of Calgary, Calgary, AB, Canada; 4 Department of Medicine, McGill University, Montreal, QC, Canada; 5 Department of Medicine, University of Alberta, Edmonton, AB, Canada; 6 Department of Medicine, University of Toronto, Toronto, ON, Canada; 7 Institute for Clinical Evaluative Sciences, Toronto, ON, Canada

## Abstract

**Background:**

Stroke has emerged as a significant and escalating health problem for Asian populations. We compared risk factors, quality of care and risk of death or recurrent stroke in South Asian, East Asian and White patients with acute ischemic and hemorrhagic stroke.

**Methods:**

Retrospective analysis was performed on consecutive patients with ischemic stroke or intracerebral hemorrhage admitted to 12 stroke centers in Ontario, Canada (July 2003-March 2008) and included in the Registry of the Canadian Stroke Network database. The database was linked to population-based administrative databases to determine one-year risk of death or recurrent stroke.

**Results:**

The study included 253 South Asian, 513 East Asian and 8231 White patients. East Asian patients were more likely to present with intracerebral hemorrhage (30%) compared to South Asian (17%) or White patients (15%) (p<0.001). Time from stroke to hospital arrival was similarly poor with delays >2 hours for more than two thirds of patients in all ethnic groups. Processes of stroke care, including thrombolysis, diagnostic imaging, antithrombotic medications, and rehabilitation services were similar among ethnic groups. Risk of death or recurrent stroke at one year after ischemic stroke was similar for patients who were White (27.6%), East Asian (24.7%, aHR 0.97, 95% CI 0.78-1.21 vs. White), or South Asian (21.9%, aHR 0.91, 95% CI 0.67-1.24 vs. White). Although risk of death or recurrent stroke at one year after intracerebral hemorrhage was higher in East Asian (35.5%) and White patients (47.9%) compared to South Asian patients (30.2%) (p=0.002), these differences disappeared after adjustment for age, sex, stroke severity and comorbid conditions (aHR 0.89 [0.67-1.19] for East Asian vs White and 0.99 [0.54-1.81] for South Asian vs. White).

**Conclusion:**

After stratification by stroke type, stroke care and outcomes are similar across ethnic groups in Ontario. Enhanced health promotion is needed to reduce delays to hospital for all ethnic groups.

## Background

There is an emerging epidemic of acute stroke in Asian populations, with recent data suggesting that the prevalence of stroke has increased 3 to 5 fold in the last 40 years in South Asian patients [1] whereas stroke rates have plateaued or declined in general North American populations [2]. The Sino-MONICA-Beijing stroke study also reported a higher incidence of stroke, particularly hemorrhagic stroke, in China compared to other countries [3]. Deaths due to stroke are higher in South Asian and East Asian groups compared with White patients in the UK and Canada [4,5]. Thus, there is a need to better understand ethnic differences in stroke, especially since South Asian and East Asian groups are among the fastest growing populations in the world [6].

Most studies have identified a strikingly higher prevalence of diabetes, hyperlipidemia, and hypertension in Asian populations with stroke compared to Whites, possibly accounting for the increased burden of stroke in Asian populations [1,7-11]. Although there is growing evidence of inter-ethnic differences in risk factors for developing stroke, there are sparse data on potential ethnic differences in stroke characteristics, quality of stroke care or outcomes. Existing evidence is further limited by incomplete ascertainment of risk factors, stroke and stroke type, and by studies derived from single centers and/or lacking direct comparisons between ethnic groups [1,7,8,11-13]. The aim of this study was to compare stroke characteristics, delivery of acute stroke care and risk of death or hospitalization for recurrent stroke in South Asian (those originating from India, or Pakistan), East Asian (originating from China, Japan, Korea, Vietnam, Thailand or Laos) and White patients with acute ischemic or intracerebral hemorrhagic stroke, using a large clinical database of patients from in 12 stroke centers in Ontario, Canada.

## Methods

### Data sources

We conducted a retrospective analysis of clinical data from the Registry of the Canadian Stroke Network (RCSN) from July 2003 to March 2008 in Ontario, Canada. The RCSN collects data from 12 stroke centers representing different geographic regions within the province of Ontario, Canada. Ontario has a population of 11.5 million people and contains 63% of persons of South Asian descent (794,000), and 47% of persons of East Asian descent (686,000) residing in Canada [6]. The registry prospectively identifies all consecutive patients with a diagnosis of acute stroke or transient ischemic attack seen in the emergency department or admitted to hospital [14]. Trained neurology research nurses collect data at each site using chart abstraction and care provider interview when needed. Detailed clinical data are collected, including demographics, stroke type, severity, clinical presentation including the Oxfordshire Community Stroke Project classification scheme for ischemic stroke [15], time intervals between stroke onset and the arrival to hospital, brain imaging, in-hospital treatment, medication prescriptions, in-hospital investigations, and discharge status. Inter-rater reliability of chart abstraction for key variables in 120 patients was substantial to excellent (kappa 0.66-1.0) [14].

The RCSN database is housed at the Institute for Clinical Evaluative Sciences, where it is linked to population-based administrative databases using unique encrypted patient identifiers. We used the Ontario Registered Persons Database to determine one-year mortality after stroke, and used the Canadian Institute for Health Information Discharge Abstract Database to identify readmissions for stroke. The Registered Persons Data Base is updated daily and includes all out of hospital deaths regardless of location within the province. The Discharge Abstract Database contains up to 16 diagnosis fields for all patients admitted to Ontario hospitals and uses the coding system of the International Classification of Diseases, 10^th^ revision. We used ICD-10 codes I61-I62, I63.3-I63.5, I63.8, I63.9, I64 with a positive predictive value of 0.85-0.98 to identify recurrent hospitalization for stroke [16].

### Patient population

We identified all patients aged 20 years or older with a diagnosis of acute ischemic stroke or acute intracerebral hemorrhage (ICH). Ascertainment of stroke cases in the RCSN is based on clinical presentation and findings on computed tomography (CT) or magnetic resonance imaging (MRI) of the brain. Patients with undetermined stroke type, in-hospital or recurrent strokes were excluded.

Patients were included in the study if their ethnicity was specified as East Asian (originating from China, Japan, Korea, Vietnam, Thailand or Laos), South Asian (originating from Pakistan or India) or White. Ethnicity was determined by research nurses from chart review using recorded ethnicity (and not from interpretation of surnames) and in some cases, interviews with the care providers using the above a priori ethnic categories. Patients of other ethnic categories including Hispanic, Filipino, Black and Native Indian (3% of total), were not included because of small sample sizes. Patients with mixed race were classified as ‘other’. To maintain a high specificity of the ethnic categories, any individual without an ethnic categorization (40% of registry patients) were not included in the analysis.

### Study variables

Prognostic characteristics at baseline for this analysis included clinical variables from the Charlson Comorbidity index (previous history of myocardial infarction, heart failure, cerebrovascular disease, peripheral arterial disease, renal impairment, cancer, dementia, chronic pulmonary disease, hypertension, rheumatic disease, peptic ulcer disease, liver disease, hemiplegia, metastatic carcinoma and AIDS/HIV), atrial fibrillation, and smoking that have been shown to be predictive of mortality or recurrent stroke in patients with stroke [17-20]. Initial stroke severity was assessed using the validated Canadian Neurological Scale (CNS) [21]. Lower CNS scores correspond to greater stroke severity. Patients were recorded as “unconscious” when the clinical notes indicated that they were unresponsive or in coma at presentation. We also examined socioeconomic status (SES) using area level median household income, based on residential postal code and the 2001 Canadian Census.

Quality of care measures included the following processes of stroke care delivery: rates of thrombolysis, stroke unit admission, stroke team assessment (a multidisciplinary group of stroke specialists including physicians, nurses, occupational therapists, physiotherapists, and speech language pathologists), carotid imaging, echocardiography, and use of antithrombotic therapy.

### Statistical analysis

Baseline characteristics, processes of care, and clinical outcomes were stratified by stroke type and compared across ethnic groups using Chi-square testing or ANOVA where appropriate. To determine the independent association of ethnicity on time to death or recurrent stroke at one year, we first stratified by stroke type (ischemic or ICH) then risk adjusted for prognostic covariates (age, sex, SES, Charlson comorbidity index score, CNS score, history of atrial fibrillation, smoking status, and admission to stroke unit) using Cox proportional hazards models. Secondary analyses evaluated the outcomes of death at 30 days and one year and rates of discharge home after the acute stroke admission. Analysis of deviance residuals was used to test for possible violations of the proportional hazards assumption for all Cox models. Assumptions for logistic regression modeling were met. P-values were 2 sided. Statistical significance was defined with a criterion of p<0.05. All analyses were performed with SAS statistical software version 9.1 (SAS Institute Inc., Cary, NC). The institutional ethics review board of Sunnybrook Health Sciences Centre and the publications committee of the Registry of the Canadian Stroke Network approved this study.

## Results

After exclusions (720 in-hospital strokes, 925 recurrent strokes, 439 undetermined stroke), 8,997 patients were identified with first acute stroke, with 253 categorized as South Asian, 513 as East Asian and 8231 as White. Ischemic stroke was more common than ICH for all ethnic groups, occurring in 70% of East Asian, 83% of South Asian and 85% of White patients. East Asian patients were more likely to present with ICH (29.6%) compared to South Asian (17%) or White patients (14.7%) (p<0.001).

### Risk factors and stroke characteristics

In the cohort with ICH, South Asian patients were younger and more likely to be male, to reside in a low income neighborhood and to have hyperlipidemia compared to White or East Asian patients, while White patients were more likely to smoke cigarettes, consume more than two alcoholic drinks per day and have a Charlson comorbidity index score of greater than two (Table 1). There was no significant difference in baseline blood pressure or prevalence of hypertension among ethnic groups. However, East Asian patients had the lowest median CNS score indicating greater stroke severity compared to South Asian or White patients.

**Table 1:** Baseline characteristics according to ethnicity for patients with ICH, %

**Characteristic**	**East Asian N=152**	**South Asian N=43**	**White N=1214**	**p value**
Age, mean (SD) y	62 .0 (17.6)	54.1 (15.1)	64.2 (15.8)	<0.001
Female	49.8	41.9	53.5	0.09
Married	60.5	73.0	59.3	<0.001
Living alone	6.9	<5%	16.1	<0.001
Median income quintile				
Q1 (low income)	27.7	33.8	20.8	<0.001
Q2	31.3	21.6	20.5	
Q3	23.2	18.9	19.3	
Q4	18.0	13.5	18.2	
Q5 (high income)	11.2	8.1	20.4	
Previously independent	51.5	56.8	47.1	0.02
Smoker (current)	6.9	<5	14.4	0.001
Alcohol (>2 drinks/day)	<5	<5	3.9	0.008
Diabetes mellitus	14.6	14.9	10.0	0.04
Hypertension	37.8	32.4	35.2	0.62
Hyperlipidemia	10.3	20.3	14.3	0.08
Family history of stroke	4.7	<5	2.7	0.2
Previous stroke or TIA	11.2	<5	11.2	0.33
Charlson score (>2)	16.9	13.6	24.4	<0.001
Systolic blood pressure mean, (SD), mmHg	168.3 (37.7)	170.5 (43)	172.2 (36)	0.6
Diastolic blood pressure mean, (SD), mmHg	92.7 (20.9)	91.8 (23.5)	90.1 (21.6)	0.2
LOC on arrival				
Alert	44.7	58.1	55.2	0.09
Drowsy	29.6	27.9	25.5	
Unconscious	23.7	14.0	18.8	
CNS score (median)	5.0	8.0	6.5	0.005
CNS score >8	33.5	48.0	41.6	0.08

*Abbreviations*: *TIA* transient ischemic attack, *LOC* level of consciousness, *CNS* Canadian Neurologic Scale.

In the cohort with ischemic stroke, South Asian patients again had a lower mean age and were more likely to be male, have hypertension, diabetes and dyslipidemia compared to other ethnic groups, while White patients were more likely to live alone, smoke cigarettes, have atrial fibrillation and higher Charlson comorbidity scores (Table 2). Blood pressure on admission was higher in South Asian patients than in White or East Asian patients. Severity of stroke was slightly greater in South Asian and East Asian patients compared to White patients. There were differences in stroke presentation, with East Asian patients more likely to present with lacunar syndromes and total anterior circulation syndromes and less likely to present with TIA compared to other ethnic groups.

**Table 2:** Baseline characteristics for patients with ischemic stroke, %

**Characteristic**	**East Asian N=361**	**South Asian N=210**	**White N=7017**	**p value**
Age, mean (SD) y	72.0 (14.4)	69.1 (12.8)	72.3 (13.7)	<0.001
Female	51.0	40.5	47.8	0.051
Married	61.2	62.9	52.8	<0.001
Living alone	6.9	8.1	22.8	<0.001
Median income quintile				
Q1 (low income)	26.0	31.4	21.4	<0.001
Q2	23.3	21.4	21.3	
Q3	17.2	19.5	18.2	
Q4	16.3	13.8	17.9	
Q5 (high income)	16.1	12.4	19.7	
Previously independent	77.0	76.2	80.0	0.2
Smoker (current)	12.5	8.1	19.5	<0.001
Alcohol (>2 drinks/day)	1.9	<5	6.2	<0.001
Diabetes mellitus	26.3	41.9	24.5	<0.001
Hypertension	72.0	74.8	67.8	0.03
Dyslipidemia	30.7	42.4	34.4	0.02
Family history of stroke	7.8	9.5	9.6	0.5
Peripheral arterial disease	2.5	4.3	6.9	0.002
Atrial fibrillation	13.3	9.0	17.4	<0.001
Previous myocardial Infarction	6.6	15.7	15.0	<0.001
Previous stroke or TIA	28.8	30.5	32.0	0.4
Charlson score (>2)	19.9	30.5	36.8	<0.001
Systolic blood pressure mean, (SD), mmHg	158.1 (29.7)	164 (32.3)	159.1 (30)	0.04
Diastolic blood pressure mean, (SD), mmHg	83.8 (16)	84.6 (16.8)	83 (17)	0.049
LOC on arrival				
Alert	79.8	85.7	87.8	<0.001
Drowsy	16.3	10.5	9.7	
Unconscious	2.8	2.9	2.2	
CNS score (median)	8.5	8.5	9	<0.001
CNS score >8	53.3	57.0	58.7	0.1
Oxford Community Stroke Project				
Lacunar syndrome	23.9	18.6	16.1	0.01
Partial anterior	35.2	40.2	39.3	
Posterior circulation	20.6	20.1	23.2	
Total anterior circulation	13	9.3	11.0	
TIA	5.4	7.8	6.0	

*Abbreviations*: *TIA* transient ischemic attack, *LOC* level of consciousness, *CNS* Canadian Neurologic Scale.

### Processes of stroke care

Time from symptom onset to hospital arrival was delayed in all ethnic groups with nearly half of all patients arriving more than 6 hours after stroke symptom onset (Table 3). Among those admitted to hospital (85% of East Asian, 79.4% of South Asian and 85% of White patients), East Asian patients were more often admitted to the ICU. However, this difference was not evident when stroke types were examined individually. Use of CT scan, carotid imaging, echocardiography, prescribing of antithrombotic medications, and referral to rehabilitation services were similar between the groups. Among patients with ischemic stroke arriving within 2 hours of symptom onset, East Asian and South Asian patients were more likely than White patients to receive thrombolytic therapy.

**Table 3:** Emergency department and hospital processes of care for ischemic stroke and ICH, %

**Characteristic**	**East Asian N=513**	**South Asian N=253**	**White N=8231**	**p value**
Time to arrival to ER				
Median time (h)	5.97	4.9	5.1	0.3
> 6 hours	49.9	47.0	47.3	0.09
2-6 hours	20.1	23.7	18.7	
<2 hours	30.0	29.3	34.0	
CT scan	99.4	99.2	99.3	0.9
Admission to				
Stroke unit	42.2	53.7	51.2	<0.001
ICU	14.9	11.9	11	
Stroke team consult	77.8	82.6	78	0.3
Neurosurgery consult	84.6	88.1	84.1	0.3
Occupational therapy	77.5	77.6	78.2	0.9
Physiotherapy	81.4	81.6	82.1	0.9
Speech therapy	64.9	62.7	61.1	0.3
**Ischemic stroke**				
tPA in those presenting within 2hrs	48.5	44.8	36	0.02
Carotid imaging	78.7	77.6	79.4	0.8
Echocardiography	67	69	71.2	0.2
ASA*	92	90.9	92.5	0.7
Warfarin for atrial fibrillation*	64.4	65.4	69.4	0.7

*Among those alive at discharge.

### Prognosis

**Figure 1:**
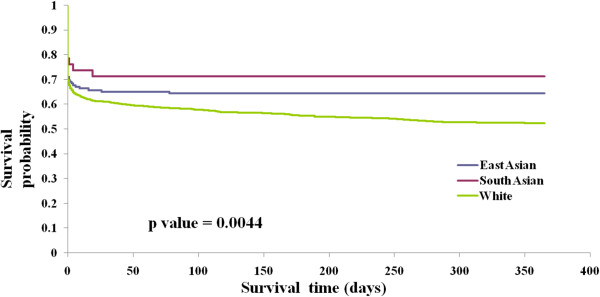
Kaplan Meier survival curve for one-year risk of death or recurrent stroke in ICH patients by ethnicity.

**Figure 2:**
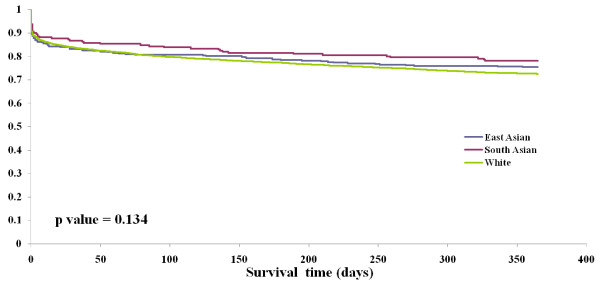
Kaplan Meier survival curve for one-year risk of death or recurrent stroke in ischemic stroke patients by ethnicity.

By one year, 43.9% of patients with ICH and 22.2% of patients with ischemic stroke had died. In the cohort with ICH, both one-year mortality and the combined endpoint of one-year mortality or recurrent stroke admission were lower in South Asian and East Asian patients compared to White patients (Table 4, Figure 1). However, this was no longer significant after adjustment for baseline differences (Table 5). In the cohort with ischemic stroke, East Asian patients were less likely than White patients to be discharged home (adjusted HR 0.70; 95% CI 0.54 to 0.90), however, there were no significant differences in 30-day mortality, 1-year mortality or risk of death or recurrent stroke among ethnic groups (Figure 2, Tables 4 and 5).

**Table 4:** Unadjusted outcomes according to ethnicity

**Outcomes**	**East Asian**	**South Asian**	**White**	**p value**
**Intracerebral hemorrhage**	N= 152	N=43	N=1214	
Discharged home* %	27.8	45.5	28.5	0.1
Mortality at 30 days %	32.9	27.9	35.4	0.5
Mortality at one year %	35.5	30.2	45.4	0.01
Mortality or recurrent stroke at one year %	35.5	30.2	47.9	0.002
**Ischemic stroke**	N=361	N=210	N=7017	
Discharged home* %	43.1	52.8	49.7	0.04
Mortality at 30days %	10.2	9.5	12.0	0.3
Mortality at one year %	19.9	19.0	22.4	0.3
Mortality or recurrent stroke at one year %	24.7	21.9	27.6	0.1

*Among those alive at discharge.

**Table 5:** Adjusted outcomes according to ethnicity*

	**East Asian vs. White**		**South Asian vs. White**	
	HR* (95%CI)	**p-value**	HR* (95%CI)	**p-value**
**Intracerebral hemorrhage**				
Discharged home vs. other**	0.94 (0.53 to 1.65)	0.8	1.29 (0.54 to 3.1)	0.6
Mortality at 30 days	1.07 (0.79 to 1.45)	0.6	1.3 (0.69 to 2.46)	0.4
Mortality at one year	0.92 (0.69 to 1.22)	0.5	1.07 (0.58 to 1.96)	0.8
Mortality or recurrent stroke at one year	0.89 (0.67 to 1.19)	0.4	0.99 (0.54 to 1.81)	0.9
**Ischemic stroke**				
Discharged home vs. other**	0.70 (0.54 to 0.90)	0.006	1.07 (0.77 to 1.49)	0.7
Mortality at 30 days	0.81 (0.58 to 1.14)	0.2	0.94 (0.59 to 1.48)	0.8
Mortality at one year	0.94 (0.74 to 1.2)	0.6	1.02 (0.74 to 1.4)	0.9
Mortality or recurrent stroke at one year	0.97 (0.78 to 1.21)	0.8	0.91 (0.67 to 1.24)	0.6

* Hazard ratios are adjusted for age, sex, area level median income, Charlson comorbidity index score, CNS score, history of atrial fibrillation, smoking status, and admission to stroke unit.

**Odds ratio among those discharged alive.

## Discussion

In this large cohort of stroke patients with detailed socio-demographic and clinical information, we found substantial differences in risk factors and stroke characteristics among those identified as East Asian, South Asian and White ethnic groups. However, processes of stroke care delivery and stroke outcomes were similar among ethnic groups.

Our finding of more frequent and more severe ICH in East Asian patients compared to White or South Asian patients is consistent with previous studies [11-13,22]. It has been speculated that higher rates of hemorrhagic stroke in East Asian patients is related to a greater prevalence of hypertension and a greater propensity to experience hemorrhagic stroke at lower systolic blood pressure levels than other Western populations [23,24]. In our study, both a pre-stroke history of hypertension and baseline systolic blood pressure on presentation were similar among ethnic groups, while alcohol intake – another potential risk factor for hemorrhagic stroke – was lower in East Asian compared to other ethnic groups. South Asian patients with ICH were 10 years younger on average compared to White or East Asian patients, but tended to have strokes of lesser severity than those seen in White or East Asian patients. This younger age at presentation with ICH in the South Asian patients is consistent with other stroke studies [7,25]. Our study found that baseline risk factors that could be associated with premature strokes, such as hypertension, dyslipidemia and diabetes, were more common in South Asian compared to other ethnic groups. However, other risk factors for ICH were paradoxically favorable in South Asian patients –fewer comorbid conditions overall, less smoking, and lower alcohol intake.

In our cohort with ischemic stroke, we also found that South Asian patients had a significantly higher prevalence of diabetes, dyslipidemia, and hypertension compared with other ethnic groups. This constellation of disproportionately elevated vascular risk factors is consistent with other studies of heart disease and ischemic stroke in South Asian populations [7-10,25-27]. Our findings of a higher prevalence of atrial fibrillation, alcohol consumption, and tobacco use in White patients with ischemic stroke are also consistent with other reports [1,7].

Ensuring a high standard of pre-hospital and hospital stroke care regardless of ethnicity is a critical goal for health care systems. In our study, all ethnic groups studied had significant delays in hospital arrival raising concern for a lack of recognition of stroke symptoms, failure to understand stroke as an emergency, or poor access to transportation. Despite differences in socioeconomic status, South Asian and East Asian patients were just as likely or more likely to receive appropriate emergency and in-hospital stroke management as well as secondary stroke prevention. East Asian patients were more likely admitted to the ICU. This was likely due to their higher occurrence of hemorrhagic strokes and lower levels of consciousness on admission. These findings are in contrast to two single center studies that found South Asian patients were less likely to receive lipid lowering therapy and East Asian patients were less likely to receive antiplatelet agents [7,10]. The lack of ethnic differences in quality of care in our study may reflect inter-regional/ inter-institutional differences or that our cohort was derived from multiple specialized stroke centers within an organized regional system of stroke care and in the context of a universal health care system.

Despite differences in baseline risk factors, short term and long term prognosis after ICH and ischemic stroke was generally similar among the identified ethnic groups. This extends the work by previous groups that only examined short- term outcomes in East Asian patients [8] and death certificate analyses for hemorrhagic stroke [28]. Previous studies reported conflicting findings with one study observing increased 30-day mortality in South Asian patients with ischemic stroke [29], and two studies demonstrating lower 28 day mortality in South Asian patients for all stroke [7] and lower nine-month mortality in Asian patients with ischemic stroke compared to White patients [30]. However, these studies had limited adjustment for key prognostic factors or did not separate different types of Asian patients despite significant differences in major Asian subgroups.

The strengths of this study include the rigorous case ascertainment of stroke using established and uniform definitions, the multi-center, multi-ethnic cohort and an extensive adjustment for confounding prognostic factors. However, there are several limitations to be noted. First, the stroke registry only had ethnicity recorded for 60% of patients and thus, these results need to be confirmed in population-based studies. However, this analysis still represents one of the largest clinically detailed evaluations of ethnic differences in stroke care and long term prognosis in these populations. Second, our cohort comprised patients seen at urban tertiary stroke centers with neurologist and CT scanning capability therefore, our results may not be generalized to settings without these capabilities. Finally, we were unable to adjust for year of immigration or generation status. However, given that the largest Asian immigration waves in Canada were in the last 35 years [6], our cohort is likely mostly comprised of first generation immigrants.

## Conclusion

Stroke is increasing dramatically in East Asian and South Asian populations globally, such that considerable efforts should be placed on stroke prevention in these groups. Our study found that stroke risk factors and stroke type differ significantly among those patients identified as East Asian, South Asian and White ethnic groups in our cohort. Future population based studies confirming these findings and evaluating the incidence of stroke subtypes among the ethnic groups are needed. Key future strategies may include improving public health programming to reduce delays in hospital arrivals for all ethnic groups, targeting East Asian patients for the prevention of hemorrhagic stroke and targeting young South Asian patients to control blood pressure and dyslipidemia. Further studies exploring the underlying mechanisms to address the higher burden of ICH in East Asian patients and premature ICH onset in South Asian patients are needed. Our findings of similar stroke care and prognosis between ethnic groups are reassuring but should be confirmed in centers without specialized stroke teams.

## Competing interests

The authors declare that they have no conflict of interest. The institutional ethics review board of Sunnybrook Health Sciences Centre and the publications committee of the Registry of the Canadian Stroke Network approved this study.

## Authors’ contributions

NK, HQ, MDH, MKK conceived the concept and design of the study. LZ, HZ performed the statistical analysis. NK, HQ, AP, MDH, MKK, LZ, HZ, LP, FM, BS interpreted the results. NK, HQ, MDH, MKK, LZ, HZ, LP, AP, FM, and BS helped to draft the manuscript. All authors read and approved the final manuscript.

## Pre-publication history

The pre-publication history for this paper can be accessed here:

http://www.biomedcentral.com/1471-2377/13/74/prepub
